# Grasping actions and social interaction: neural bases and anatomical circuitry in the monkey

**DOI:** 10.3389/fpsyg.2015.00973

**Published:** 2015-07-14

**Authors:** Stefano Rozzi, Gino Coudé

**Affiliations:** Department of Neuroscience, University of Parma, Parma, Italy

**Keywords:** motor, mirror neurons, intention, motor goal, grasping, parietal

## Abstract

The study of the neural mechanisms underlying grasping actions showed that cognitive functions are deeply embedded in motor organization. In the first part of this review, we describe the anatomical structure of the motor cortex in the monkey and the cortical and sub-cortical connections of the different motor areas. In the second part, we review the neurophysiological literature showing that motor neurons are not only involved in movement execution, but also in the transformation of object physical features into motor programs appropriate to grasp them (through visuo-motor transformations). We also discuss evidence indicating that motor neurons can encode the goal of motor acts and the intention behind action execution. Then, we describe one of the mechanisms—the mirror mechanism—considered to be at the basis of action understanding and intention reading, and describe the anatomo-functional pathways through which information about the social context can reach the areas containing mirror neurons. Finally, we briefly show that a clear similarity exists between monkey and human in the organization of the motor and mirror systems. Based on monkey and human literature, we conclude that the mirror mechanism relies on a more extended network than previously thought, and possibly subserves basic social functions. We propose that this mechanism is also involved in preparing appropriate complementary response to observed actions, allowing two individuals to become attuned and cooperate in joint actions.

## Introduction

Over the last 50 years, sensorimotor neuroscience has produced an extensive body of work dedicated to the study of grasping. The motor act of grasping is multifaceted and lies at the crossroad between action and perception. Here, a distinction should be drawn between the grasping motor act and the action of grasping. A grasping motor act can be defined as a series of joint movements, like clasping the fingers on an object, aimed at achieving the motor goal of seizing. An action of grasping consists in a sequence of fluently linked motor acts that altogether are aiming at the achievement of an overarching behavioral goal. For instance, a grasping action would consist in reaching, grasping a fruit and bringing it to the mouth for eating. Under normal circumstances, a grasping motor act is executed within a sequence of other motor acts together forming a grasping action. Such an action can be driven by a wide gamut of needs and aimed at a variety of overarching goals such as feeding, exploring the environment, or interacting with other individuals. Interestingly, socially appropriate behaviors require a continuous monitoring of the social environment. Accordingly, numerous studies both on monkey and human focused on analyzing the motor behavior, especially grasping actions, to investigate basic social interactions. Altogether, these studies demonstrate that grasping is modulated by the social context in which it occurs. This, in turn, implies that the motor system, that actually produces the behavior itself, is involved in a larger network encoding social aspects of real life. However, the neural mechanisms at the basis of this coupling between social cognition and motor behavior have not yet been fully unveiled. In this review, we describe the basic neural mechanisms underpinning grasping and show how these same mechanisms are also at the bases of cognitive abilities that are basic aspects of social cognition such as action understanding and intention reading. This paper mainly focus on the anatomical and functional literature based on the macaque monkey model. Indeed, monkeys have been used for brain studies since the beginning of the twentieth century (Brodmann, 1909; Bucy, 1933, 1935; Fulton, 1935), and we owe to monkey studies a huge part of our knowledge on the neuroanatomy and neurophysiology of the motor system. This is especially true for the neural bases and the anatomical circuitry involved in grasping. Monkeys are capable of using their hands for grasping in a way that is very similar to humans. Evolution is opportunistic and conservative: working mechanisms tend to be retained through generations and species and novelty tends to be built by adapting extant material and processes to the new demands. Without denying the obvious gap existing between the monkey brain and the human brain (see Iriki and Sakura, 2008; Passingham, 2009), we think that the macaque model remains invaluable for the anatomo-physiological study of grasping. Thus, in the first part of the paper we describe the anatomical structure of the motor cortex in the monkey and the cortical and sub-cortical connections of the areas forming it. In the second section, we review the monkey functional literature showing two important aspects: (1) that motor neurons are not only involved in movement execution, but also in the sensory-motor transformation for grasping, and (2) that a population of these neurons encodes the goal of grasping motor acts and the motor intention behind action execution. In the third part, we describe one of the mechanisms—the mirror mechanism—considered to be at the basis of action understanding and intention reading. In particular we discuss how important aspects of the social environment such as the spatial representation of self, objects and others, modulate the motor and mirror neurons activity, influencing monkeys behavioral responses. In the last part, we briefly show that mechanisms similar to those described in the monkey are also present in the homo species.

## Anatomy of the Motor System

### The Motor Cortex: General Organization

At the end of the nineteenth century the general view of the organization of the motor system was that the movements were controlled by subcortical centers, while the cerebral cortex was involved cognitive functions. This view was challenged by pioneering studies demonstrating that the electrical stimulation of a specific part of the frontal cortex (motor cortex) evoked body movements in different species of animals (Fritsch and Hitzig, 1870; Ferrier, 1873; see Porter and Lemon, 1993). The idea that the motor cortex contains a simple map of the muscles was in line with Jackson’s observations on the epileptic seizures in human patients. At the beginning of the twentieth century, Campbell (1905) identified a possible anatomical substrate accounting for Jackson’s observations in his architectonic map of the human cerebral cortex. Campbell’s (1905) view was that the precentral cortex was implicated in motor control, while the intermediate sector was involved in what will be later called “higher order motor functions.” A similar view emerged from Brodmann’s (1909) work, where he confirmed the existence of two motor areas, area 4 and area 6, and provided a more detailed map of the frontal lobe both in monkeys and humans. The idea that architectonic differences reflects functional specificity was later supported by Fulton (1935), who showed that the ablation of area 6 produces specific deficits in the execution of skilled movements. This observation led him to refer to this region as *premotor cortex*. However, a few years later, Woolsey’s electrophysiological studies (Woolsey and Settlage, 1952) casted doubts about the existence of a high order motor area rostral to area 4, and led him to conclude that area 4 and posterior area 6 form together a functional entity, while the not electrically excitable rostral area 6 does not belong of the motor cortex.

**FIGURE 1:**
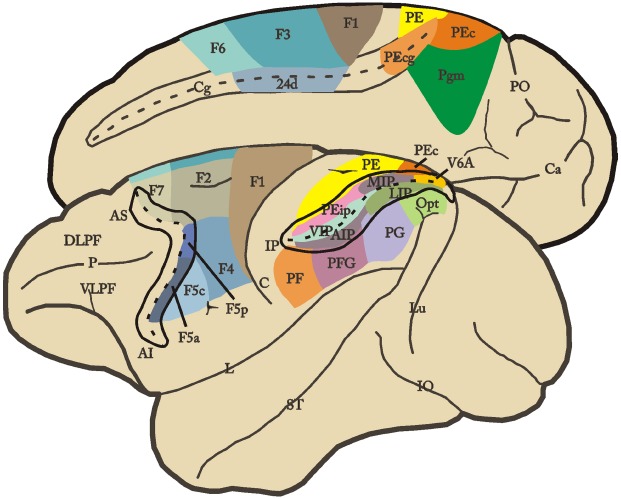
**Lateral and mesial views of the monkey brain showing the parcellation of the agranular frontal and posterior parietal cortex.** Intraparietal, arcuate and cingulated sulci are shown unfolded. For the nomenclature and definition of the agranular frontal and posterior parietal areas, see text. AI, inferior arcuate sulcus; AS, superior arcuate sulcus; C, central sulcus; Ca, calcarine fissure; Cg, cingulated sulcus; DLPF, dorsolateral prefrontal cortex; IO, inferior occipital sulcus; L, lateral fissure; Lu, lunate sulcus; P, principal sulcus; PO, parieto-occipital sulcus; ST, superior temporal sulcus; VLPF, ventrolateral prefrontal cortex.

Brodmann’s definition of area 6 as a single architectonic entity was also challenged by subsequent anatomical studies in which this sector was divided in different areas (e.g., Vogt and Vogt, 1919; Von Bonin and Bailey, 1947; Barbas and Pandya, 1987). Recently, a more objective assessment of areal borders was provided by combining cytoarchitectonic and neurochemical techniques (see Geyer et al., 2000; Belmalih et al., 2007). This multiarchitectonic approach yielded a more refined map of the motor cortex of the macaque monkey (Figure 1; Matelli et al., 1985, 1991; Belmalih et al., 2009). In this parcellation, area F1 roughly corresponds to Brodman’s area 4 (primary motor cortex), whereas the mesial, dorsal, and ventral sectors of Brodman’s area 6 are each divided into caudal and rostral areas. This parcellation has been further validated by converging functional evidence showing that each of these architectonic subdivisions are connectionally and functionally distinct. The resulting map indicates that the macaque motor cortex is a mosaic of distinct areas and contains a multiplicity of body movement representations, each emphasizing different categories of behavior and playing a specific role in motor control (see Rizzolatti et al., 1998). Thus, the “mapping from cortex to muscles is not fixed, as was once thought, but instead is fluid, changing continuously on the basis of feedback in a manner that could support the control of higher-order movement parameters” (Graziano, 2006).

### Connections of the Motor Areas of the Monkey

Connectional studies are warranted to gather clues about their functional role and complete the picture etched through architectonic studies. By means of tract tracing studies, it has been shown that each motor area is characterized by a specific pattern of connections. Based on these general connectivity patterns, the premotor areas have been grouped into two major classes (Rizzolatti and Luppino, 2001): the caudal (F2, F3, F4, F5p, and F5c) and the rostral (F5a, F6, and F7) premotor areas. In the following paragraphs, we describe the descending and cortical connections of these motor areas, and draw hypotheses on their possible functional role.

#### Descending Motor Pathways and Intrinsic Motor Connections

**FIGURE 2:**
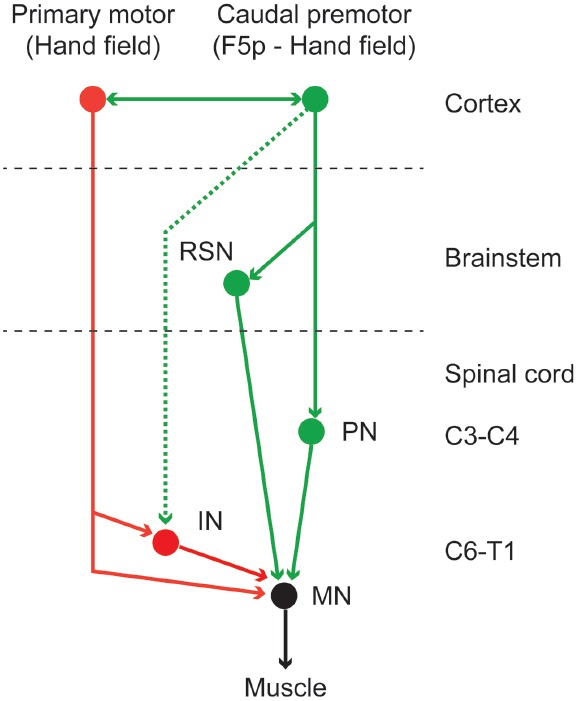
**Schematic view of the descending pathways involved in the control of hand grasping.** Caudal premotor areas are involved in generating and controlling hand grasping movements not only through the primary motor cortex, but also in parallel with it. The red lines indicate the corticospinal projection from the primary motor cortex to the cervical enlargement of the spinal cord. This is the only pathway directly accessing the spinal motor neurons (see Lemon, 2008). The green solid lines indicate the descending projection from the caudal premotor area F5p (Borra et al., 2010) to the reticulo-spinal neurons and to the spinal propriospinal system (Isa et al., 2007). The corticospinal projection from caudal premotor areas to the cervical enlargement (dashed green line) are much weaker than those deriving from the primary motor cortex. IN, interneurons; MN, motoneurons; PN, propriospinal neurons; RSN, reticulo-spinal neurons.

As a whole, the motor cortex is source of different descending motor pathways, each providing it with an access to the brainstem and spinal motor centers. Strick and coworkers (Dum and Strick, 1991; He et al., 1993, 1995) showed that the corticospinal projections are somatotopically organized and that originate both from the primary motor area and from all the caudal premotor areas. Similarly, the face and mouth cortical motor representations are sources of corticobulbar projections (Morecraft et al., 2001). The corticospinal projections mostly terminate in the intermediate zone of the spinal cord, and only F1 is source of monosynaptic projections to spinal motor neurons (Porter and Lemon, 1993). This means that F1 is the final common pathway, at the cortical level, for controlling skilled movements. However, the presence of corticospinal projections from all the caudal premotor areas clearly indicates that these areas are also involved in generating and controlling movements, not only through F1, but also in parallel with it, as also confirmed by the evidence that each of them is also somatotopically connected with F1. For example, a descending indirect pathway connecting the caudal premotor area F5p with the cervical propriospinal system was recently described and is deemed to be involved in the control of dexterous fingers movements (Sasaki et al., 2004; Isa et al., 2007; Borra et al., 2010; see also Lemon, 2008; Alstermark and Isa, 2012). Figure 2 depicts a schematic view of the descending pathways enabling hand motor control.

In contrast, none of the rostral premotor areas project directly to the spinal cord. Their descending projections reach different parts of the brainstem (Keizer and Kuypers, 1989). Furthermore, they are not directly connected with F1, and generally have a widespread pattern of connections with other motor areas. The radically different pattern of descending projection characterizing rostral and caudal areas hints to the fact that they probably are subserving different functions. The rostral areas are thought to be only indirectly involved in the generation of motor behavior through their sub-cortical projections and through their cortical connections with the caudal premotor areas.

#### Cortico-Cortical Connections

The cortical connections of the frontal motor areas involve mainly two brain regions: parietal cortex and prefrontal cortex (see Rizzolatti et al., 1998, 2014; Rizzolatti and Luppino, 2001). The connections between frontal motor and posterior parietal areas are very strong and reciprocal. Anatomical and functional evidence show that the posterior parietal cortex consists in a mosaic of areas similar to the motor cortex (Figure 1), each area is involved in processing specific aspects of sensory information and controlling different effectors (e.g., mouth, hand, arm, and eyes). In general, most IPL areas and the posterior areas of the SPL process either strictly visual or visual and somatosensory information, while the rostral areas of the SPL mainly deal with somatosensory information (Hyvärinen, 1982; Caminiti et al., 1996; Rizzolatti et al., 1997; Wise et al., 1997; Colby, 1998; Rizzolatti and Matelli, 2003; Rozzi et al., 2008). A series of largely segregated anatomical circuits linking parietal and motor areas can be identified according to the pattern of predominant connections. These circuits integrates specific motor and sensory signals and participate to particular aspects of sensory-motor transformations, and should be thus considered the functional units of the cortical motor system. The processing undertaken by these functional units results in the generation of *potential motor acts*. In the following section, we describe the anatomy and function of one of these circuits (AIP-F5), and discuss its role in transforming visual information about an object into potential motor acts appropriate to grasp it.

The second strongest source of cortical connections of the motor areas is the prefrontal cortex. Prefrontal connections primarily involve the rostral premotor areas (Barbas, 1988; Preuss and Goldman-Rakic, 1989; Luppino et al., 1993; Lu et al., 1994; Rizzolatti and Luppino, 2001; Saleem and Kondo, 2008; Gerbella et al., 2010, 2013; Borra et al., 2011). Specifically, the dorsal part of the lateral prefrontal cortex (DLPF) projects to F7, its ventral part (VLPF) projects to F5a, whereas both DLPF and VLPF project to F6. Our knowledge of the anatomo-functional organization of the prefrontal cortex is much less detailed than that of the parietal cortex. It is generally accepted that these regions are involved in “higher order” functions such as working memory, planning of actions, and motivation (see Miller and Cohen, 2001; Tanji and Hoshi, 2008). Thus, these projections could play a role in selecting the potential motor acts generated as the result of sensorimotor transformations, weighting their suitability according to context, abstract rules, memorized information, and behavioral goals. The interplay between prefrontal cortex and frontal motor areas could be at the basis of the transformation of *potential* actions into *actual* actions.

## Functional Properties of Motor Neurons: From Grasping to Intention

### Visuo-Motor Transformations for Grasping

Grasping requires the adjustment of hand conformation to the size and shape of an object. A very efficient way of to accomplish this duty has evolved in the motor system. It consists in a direct linkage between the representations of object physical features and of potential motor acts, allowing the capacity of coding objects in term of actions to execute upon them. The process of transforming object properties into corresponding potential grasping actions relies on a specific circuit linking parietal area AIP and premotor area F5. The neural properties of these areas have been widely studied. We know that area F5 contains purely motor and sensory-motor neurons, some of which responsive to the presentation of visual stimuli (Rizzolatti et al., 1988). These F5 visuo-motor neurons fall into two main classes: canonical and mirror neurons, although, recently, the additional hybrid class of “canonical-mirror” neurons has been identified (Bonini et al., 2014). In this section, we will describe the properties and the functional role of the canonical neurons.

Canonical neurons are mostly located in area F5p and discharge during the presentation of 3D objects (Murata et al., 1997; Raos et al., 2006). They have been systematically studied by means of a paradigm that allows one to separate activity related to object presentation, action preparation and action execution. For the major part, canonical neurons selectively respond to objects of a certain size, shape and orientation. Typically, their visual and motor specificity are congruent, and it was demonstrated that their activity does not depends on attention to stimuli, intention to act, or motor preparation (Murata et al., 1997). The most likely explanation for the canonical neurons discharge proposes that object presentation activates a representation of the observed object in motor format. In other words, when an object appears in the visual scene, the discharge of a specific set of canonical neurons code a *potential grasping act* congruent with the physical properties of the presented object. Note that this occurs independently of whether the act will be actually executed or not. In support of this explanation is the observation that a canonical neuron can show a visual response of the same intensity to the presentation of objects of different shape that are grasped in the same way (Murata et al., 1997; Raos et al., 2006).

As mentioned above, F5 has strong anatomical connections with the AIP area (Luppino et al., 1999; Borra et al., 2008; Gerbella et al., 2011). The functional properties of the neurons located in this area have been studied using the same paradigm adopted for the study of F5 canonical neurons (Taira et al., 1990; Sakata et al., 1995; Murata et al., 2000). By using this paradigm, AIP neurons have been divided into three classes: *motor-dominant*, *visual and motor*, and *visual-dominant* neurons. *Motor-dominant* neurons discharge during grasping either if the action is performed in light or in darkness, but do not fire during simple object fixation. *Visual-dominant* neurons discharge during grasping in light and during object fixation, but not when grasping is performed in darkness. Finally, v*isual and motor neurons* discharge stronger during grasping in light than in darkness, and also discharge during object fixation.

The evidence that AIP and F5 are nodes of a circuit involved in visuo-motor transformations for grasping was strongly supported by inactivation studies. In particular, the inactivation of either AIP (Gallese et al., 1994) or F5 (Fogassi et al., 2001) has been shown to cause important deficits in shaping the hand according to the stimulus physical characteristics during hand transport before landing on the object. Note that, once touched, the object is correctly grasped, thus showing the lack of pure motor deficits.

Several models have been proposed to explain the role of AIP and F5 in visuo-motor transformation for grasping (Taira et al., 1990; Jeannerod et al., 1995; Fagg and Arbib, 1998; Rizzolatti and Luppino, 2001; Fluet et al., 2010). Despite the fact that there is no complete agreement among these models, they share the common idea that when an object is presented, AIP neurons extract specific aspects of its intrinsic features and provide F5 with a multiple description of the possible ways to grasp it. This corresponds to what Gibson defined as *affordances* (Gibson, 1979). The lateral prefrontal cortex would activates a set of AIP and F5 neurons according to the behavioral goal, object nature, and context. Indeed, an object can be grasped with various types of grip depending not only on its physical features, but also on the different behavioral contexts. For instance, in recent studies, monkeys were trained to associate two different grip-types with corresponding color cues. The results showed that in both AIP and F5, a set of neurons were active after cue presentation, showing context-dependent grasp planning activity (Baumann et al., 2009; Fluet et al., 2010). The information about the chosen grip, according to the models, would be then sent from F5 to F1, where the movements are coded, and the final command for the execution is generated. Indeed, recent physiological experiments demonstrated that the activation of F5 is able to generate object-oriented actions through the modulation of F1 motor output (Cerri et al., 2003; Shimazu et al., 2004; Prabhu et al., 2009). Note, however, that the existence of corticospinal projections from F5p (see above; Borra et al., 2010) indicates that this area could be involved in the generation and control of movements not only through F1, but also in parallel with it (Figure 2). In particular, the F5 connections with the cervical propriospinal neurons appears to be involved in the control of dexterous fingers movements (Isa et al., 2007; Kinoshita et al., 2012). The exact functional role of these projections is still only partially understood, but evidence suggest that they may play a role in the functional recovery observed after lesions of the motor cortex. In particular, in New World monkeys, the ventral premotor hand field expands and develops new cortical connections after lesions of the primary motor cortex (Frost et al., 2003; Dancause et al., 2005, 2006; see Nudo, 2007). In macaque, it has been shown that after intensive post-lesion motor training, the ventral premotor hand field (including F5p) undergoes plastic changes and shows recovery-related increases in activity (Nishimura et al., 2007; see Nishimura and Isa, 2012).

### Coding Grasping Goal: The “Vocabulary of Motor Acts”

**FIGURE 3:**
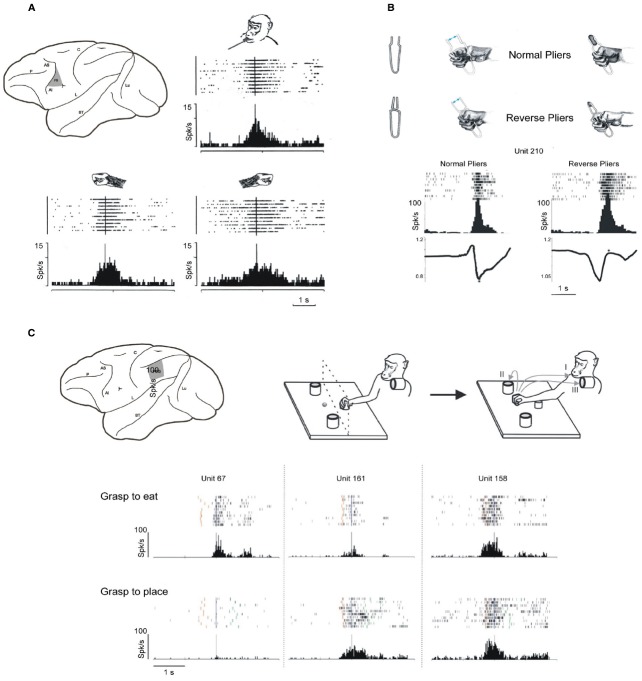
**Goal and intention encoding in areas F5 and PFG. (A)** Upper part, left: lateral view of the monkey brain showing the location of area F5; right and lower part: discharge of an F5 neuron active during grasping with the mouth, the right hand and the left hand. Raster and histograms are aligned with the moment in which the monkey touches the target object. Abscissae: time; ordinates: spikes per bin; bin width: 20 ms. Modified from Rizzolatti et al. (1988). **(B)** Example of an F5 neuron discharging during grasping with normal and reverse pliers. Upper part: pliers and hand movements necessary for grasping with the two types of pliers. Lower part: rasters and histograms of the neuronal discharge during grasping with pliers. The alignments are with the end of the grasping closure phase (asterisks). The traces below each histogram indicate the hand position, recorded with a potentiometer, expressed as function of the distance between the pliers handles. When the trace goes down, the hand closes, when it goes up, it opens. The values on the vertical axes indicate the voltage change measured with the potentiometer. Other conventions as in **(A)**. Modified from Umiltá et al. (2008). **(C)** Example of motor neuron of area PFG modulated by action intention. Upper part left: lateral view of the monkey brain showing the location of area PFG. Upper part right: paradigm used for the motor task. The monkey, starting from a fixed position, reaches and grasps a piece of food or an object, then it brings the food to the mouth and eats it (I, grasp-to-eat), or places it into a container (II/III, grasp-to-place). Lower part left: activity of three IPL neurons during grasping in the two actions. Rasters and histograms are aligned with the moment when the monkey touched the object to be grasped. Red bars: monkey releases the hand from the starting position. Green bars: monkey touches the container. Conventions as in **(A)**. Modified from Fogassi et al. (2005).

Planning and executing an *action*, such as grasping and eating an apple, implies to have an *overarching goal* (to eat the apple), to select the appropriate sequence of *motor acts*—each with its specific motor goal (reaching, grasping, bringing to the mouth, biting)—and to execute the sequence of *movements* forming each motor act (see Rizzolatti et al., 2014). Attaining action goals relies on the precise integration of the processes carried out at each of these hierarchical levels and on their accurate timing. It is well known that area F1 and F5 are mainly involved in movement implementation, and in coding the goal of motor acts, respectively. Area F5 neurons typically encode motor acts performed with the hand or the mouth (Kurata and Tanji, 1986; Gentilucci et al., 1988; Rizzolatti et al., 1988; Hepp-Reymond et al., 1994; Ferrari et al., 2003). Electrophysiological studies revealed that a large proportion of F5 neurons encode specific motor acts such as grasping or tearing, rather than simple movements (Rizzolatti et al., 1988). Typically, an F5 hand motor neuron discharge during finger movements aimed at taking possession of an object (grasping) but not during similar movements aimed at different goals (e.g., scratching). In addition, F5 neurons activates when the same goal is achieved by using different effectors/movements (e.g., taking possession either with the right hand, the left hand, or the mouth, Figure 3A). Interestingly, many neurons code specific grip types such as precision grip, finger prehension or whole hand prehension. Concerning the timing of grasping, some neurons discharge during the whole unfolding of the motor act, and others during a specific part of it (e.g., shaping of the hand).

Altogether, these data led to the proposal that F5 contains a “vocabulary” of motor acts (Rizzolatti et al., 1988). The “words” of this motor vocabulary are represented by different populations of neurons, some coding the general goal of a motor act, others coding how a specific motor act has to be executed or specifying the temporal aspects of the motor act to be executed (see Jeannerod et al., 1995). Neuroanatomical data show that F5 is densely connected with the parietal areas AIP, PF, PFG, and SII (Petrides and Pandya, 1984; Matelli et al., 1986; Cavada and Goldman-Rakic, 1989; Luppino et al., 1999; Rozzi et al., 2006; Borra et al., 2008; Gerbella et al., 2011). Areas F5 and PFG also share numerous functional properties (Leinonen et al., 1979; Hyvärinen, 1981; Rozzi et al., 2008; Bonini et al., 2010), and both contain motor neurons coding goal directed motor acts. A definitive demonstration that motor neurons indeed code motor acts has been provided by a study in which the same motor goal was achieved by employing opposite movements (Umiltá et al., 2008). Monkeys were trained to grasp objects using “normal” pliers, that is pliers that require hand closure in order to take possession of the object, and “reverse” pliers that require hand opening to achieve the same goal. The correlation between the neuron discharge and the hand movements revealed that a population of F5 neurons code goal achievement (i.e., taking possession of the target object) independently of the type of fingers movement employed (flexion or extension, Figure 3B).

### Coding Motor Intention

Based on the data described in the previous section, a dissociation seems to exist between goal and movement in the motor system. One can therefore hypothesize that some population of neurons would code an even higher level of goal representation, possibly reflecting the overarching goal of the action, and expect to find neurons discharging differently during the execution of a motor act (e.g., grasping) according to the overarching goal of the whole action (e.g., eating). Recently, a series of experiments were carried out to test this hypothesis (Fogassi et al., 2005; Bonini et al., 2010, 2012). Grasping neurons were recorded from areas PFG and F5 in two conditions: in the first condition, the monkey grasped a piece of food and brought it to the mouth for eating; in the second, the monkey grasped an object or a piece of food in order to place it into a container. Some neurons discharged stronger during grasping to eat, and weaker or did not discharge at all during grasping to place in the container. Others had an opposite behavior (Figure 3C). Note that the differential discharge occurred during the actual grasping execution, and that the grasping act itself—consisting in closing the hand on the object—was exactly the same in the two conditions. The kinematics of reaching movements, the grip force exerted, the type of object involved—metallic cube or food—or the amount of underlying motivation could not account for the differential activation of the neurons in the two conditions (Fogassi et al., 2005; Bonini et al., 2010). The discharge of these motor neurons, besides coding goals at the motor acts level, also reflects the overarching goal of the actions. Such neurons could play an important role in linking the specific motor acts belonging to an action in an appropriate motor chain, allowing the correct and fluid execution of the corresponding movement sequence. Beside this role in kinematic fluidity, their activation could have significant implications at a cognitive level. The firing of these neurons, together with that of the other neurons involved in the same action, represent the neural correlate of the overarching goal underlying the action, that is, the *motor intention* of the acting individual. Having a motor system wired as such could have been important in the phylogenetic development of the ability to read other’s intentions. One of the mechanisms possibly underlying this capacity relies on the mirror system and is be discussed in the following sections.

## The Mirror Mechanism

The discovery of the *mirror mechanism* radically changed our views on the functional role of the motor system. It is now largely accepted that the same neurons involved in motor coding can also underpin social abilities such as understanding actions, reading others’ intentions and programming contextually appropriate motor responses (see Rizzolatti et al., 2014). The existence of mirror neurons also shed light on how some basic processes involved in social cognition can be mediated by the motor system. Altogether, these functions represent fundamental aspects for social relations in primate and human societies (Sebanz and Knoblich, 2009; Bach et al., 2011; see Sebanz and Knoblich, 2008).

**FIGURE 4:**
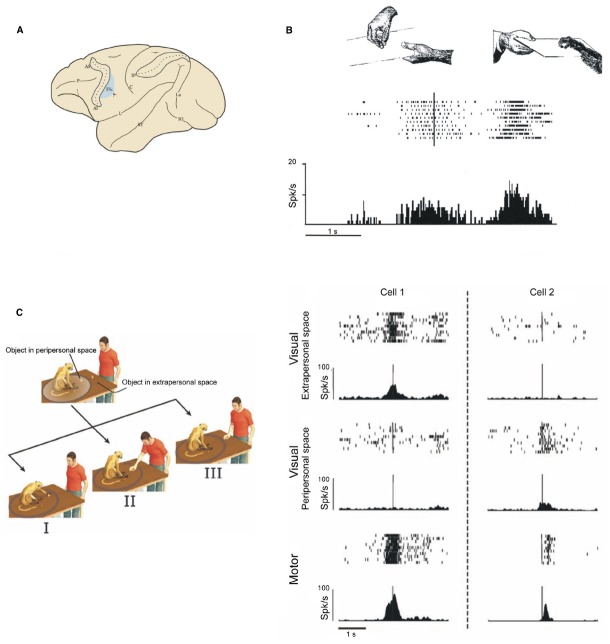
**Examples of F5 mirror neurons. (A)** Lateral view of the monkey brain showing the location of area F5c. **(B)** Mirror neuron responding during observation and execution of a hand grasping motor act. Conventions as in Figure 3A. Modified from Rizzolatti et al. (1996a). **(C)** Examples of mirror neurons whose visual response is modulated by the distance at which the observed act is performed. Left: experimental paradigm. I the monkey grasps a piece of food. II and III the experimenter grasps a piece of food located in the monkey peripersonal or extrapersonal space, respectively. Right: Cell 1: the visual response is higher during observation of grasping performed in the extrapersonal space; Cell 2: the visual response is higher during observation of grasping performed in the peripersonal space. Each panel shows a raster plot and the spike density function of the neuron response. Modified from Caggiano et al. (2009).

Simply put, mirror neurons discharge when a subject either actually performs a motor act or simply observes the same act being performed by someone else (Figures 4A,B). In other words, the observation of an action triggers in the observer’s brain a representation of that action in a motor format (Di Pellegrino et al., 1992; Gallese et al., 1996; Rizzolatti et al., 1996a; see Rizzolatti and Sinigaglia, 2010; Rizzolatti et al., 2014). The fact that an action representation in motor format can be activated by mere observation raise an important question: why don’t we automatically move when observing an action? Recent single neuron experiment showed that the activity of a significant portion of pyramidal tract neurons of area F5 is modulated by action observation (Kraskov et al., 2009), and either increase or decrease their discharge. This finding indicates that mirror neurons activity can be transmitted to the spinal cord. Considering that more than one-fourth of pyramidal tract neurons show suppression of discharge during observation, while increase firing rate during active movement, the authors suggested that this inhibitory effect might play a role in preventing movement generation during action observation.

### Mirror Neurons and Action Understanding

It has been proposed that the mirror mechanism, by matching the visual description of a motor act with its motor representation in terms of goal, allows the observer to understand what another individual is doing. Such a process would be possible because the observation of an act automatically retrieve its motor representation by tapping into the observer’s motor vocabulary (described in the previous section, see Rizzolatti and Sinigaglia, 2010; Rizzolatti and Fogassi, 2014; Rizzolatti et al., 2014). This implies that a representation of the motor goal of an act can be triggered by sensory information. The nature of the sensory information capable of activating mirror neurons has been investigated in two neurophysiological studies. In the first, mirror neurons have been demonstrated to discharge both when the monkey can fully observe an experimenter grasping an object, and when he can only see part of the action, due to its crucial part (the hand-object interaction) being hidden by a screen (Umiltá et al., 2001). Interestingly, there was no neuronal discharge if the monkey knew that there was no object to grasp behind the screen, suggesting that, in the absence of a full visual description, mirror neurons use mnemonic-contextual information to retrieve the motor representation of the observed motor act. In the second study, sensory information about the motor act was presented to the monkey in an acoustic and/or visual format. It revealed that some mirror neurons (audio-visual mirror neurons), discharge not only during the execution and the observation of a motor act producing a sound (e.g., the crackling sound of breaking a peanut), but were also activated when the monkey simply heard the sound made by the action (Kohler et al., 2002; Keysers et al., 2003). Altogether, these data indicate that mirror neurons respond to the goal of others’ motor acts also in partial or total absence of visual cues.

### Mirror Neurons and Intention Coding

Some mirror neurons share an interesting property with purely motor neurons and encode the motor intention behind the actions performed by other individuals (Fogassi et al., 2005; Bonini et al., 2010). A series of experiments was carried out to assess a possible relation between motor intention and mirror neurons activity. One way of testing this possibility was to verify whether neurons discharging during the execution and observation of grasping acts are influenced by the type of action in which the grasping acts are embedded (Fogassi et al., 2005; Bonini et al., 2010). In this purpose, grasping-related mirror neurons were recorded from parietal area PFG and premotor area F5 while the monkey executed a motor task (motor condition) and observed the same task, performed by an experimenter (visual condition). The experimental paradigm was the same as previously described in the section “coding motor” intention: an identical grasping act was embedded into two different actions, aimed at eating or placing the target in a container, respectively. The results show that in both the motor and visual condition a large proportion of mirror neurons discharged differently during the observation of the grasping act, when it was part of the two different actions (action-goal-related mirror neurons). The neuronal selectivity for the overarching goal expressed during grasping observation has been interpreted as a prediction of the action outcome. Making such a prediction is possible since the monkey knows that a given context like the type of target object or the presence of a container is followed by *grasping-to-place* action. Note, however that these neurons are not activated by contextual cues such as the observation of the target object or of the scene, but by action observation. Accordingly, it was hypothesized that when one of these neurons is activated by the observation of a grasping motor act that is part of a specific motor action (grasp-to-eat or grasp-to-place), it triggers the motor circuitry that constitutes the internal representation of the overarching goal of the sequential action. Thus, mirror neurons, besides the capacity of coding motor acts, provide individuals with a mechanism for understanding others’ intentions (Fogassi et al., 2005; Rizzolatti and Sinigaglia, 2010; Rizzolatti et al., 2014).

### Mirror Neurons and the Social Context: Space and Agency

Understanding the behavior of others’ is one of the building block of social cognition. However, in social animals in which object-oriented behaviors usually occur in the presence of other individuals, understanding the action goal and the intention behind it is not sufficient to frame this action in its social context. In this purpose, it is also very relevant to evaluate actions with respect to the position in space where they occur, and especially with respect to the observer’s position. For example, if an individual is grasping an object close to an observer, an interaction is possible. The observer has the actual possibility to interfere with the grasping action and prevent it to happen, or cooperate to it. Cooperative behaviors are common in humans, but are also documented in monkeys (Mendres and de Waal, 2000; Visalberghi et al., 2000; Visco-Comandini et al., 2015). If the mirror mechanism was only involved in action and intention understanding, the spatial location of the observed action and the vantage point of the observer would be irrelevant. However, if space also plays a role in tuning motor responses appropriate to others’ actions, as first proposed by Jeannerod (2006), these spatial factors could possibly modulate the neural discharge of mirror neurons. This hypothesis has been empirically tested in different experiments. The results showed that, although in most cases the visual response of mirror neurons is invariant with respect to spatial features, the discharge of some of them is modulated by the direction of the hand movement, the space sector (right or left) in which the motor act occurs or the hand (right or left) used by the observed agent (Gallese et al., 1996; Rozzi et al., 2008).

The effect of the distance at which an action occurs on the discharge of mirror neurons was systematically tested in a recent experiment (Caggiano et al., 2009). In this study the same motor act was executed within the monkey reaching space (peripersonal space) or outside it (extrapersonal space). About half of the studied mirror neurons discharged differently in the two conditions. Of them, 50% discharged stronger when the monkey observed the experimenter grasping a piece of food in its peripersonal space and 50% in the extrapersonal space (Figure 4C). Crucially, the authors tested whether, in these mirror neurons, space was represented in terms of a metric representation—the geometric distance between the action and the monkey—or in terms of operational representation—the pragmatic space where the monkey can actually act. To this end, a transparent barrier was introduced between the monkey and the site where the experimenter executed the action. In this condition the monkey could see the action, but was prevented from interacting with the object located within its peripersonal space. If a metric representation is at play in the mirror neurons code, peripersonal and extrapersonal space would remain unchanged, while if an operational representation occurs, the introduction of the barrier would lead to a remapping of the peripersonal into extrapersonal space. The results show that when the barrier was introduced, *extrapersonal* mirror neurons started discharging also when the observed action was performed within the peripersonal space, as if this latter were displaced far away. Taken together, these data suggest that a subpopulation of mirror neurons can code differently others’ actions depending on the space sector in which they occur. It is very likely that space location and distance are coded within the mirror neuron system in relation to the often vital possibility to interact or not with others. Thus, mirror neurons, besides being involved in action understanding, could also be important for choosing the motor response appropriate to others’ actions in their specific behavioral context.

The issue of space coding is very important also because a large number of primates actions is directed toward oneself (e.g., bringing objects to the mouth), while most of the studies about the mirror system focused on actions directed away from one’s body (e.g., reach for and grasp an object). It is well known that in the fundus of the intraparietal sulcus (area VIP) there are bimodal neurons, responding to visual and tactile stimuli, whose tactile receptive fields are located predominantly on the face and the visual receptive fields are in spatial register with the tactile ones (Colby et al., 1993; Duhamel et al., 1998). The electrical stimulation of this area evokes face movements and defensive movements of the arm toward the face (Cooke et al., 2003). Ishida et al. (2010) studied the neural properties of a population of these bimodal neurons, delimiting the extension in depth of their peripersonal space in monkeys either alone, or facing an experimenter. Typically, when a visual stimulus was presented outside the peripersonal space, at more than one meter of distance from the tactile receptive field, no visual response was recorded. However, when an experimenter was standing in front of the monkey at the same distance and a stimulus was moved close to his/her body part corresponding to the neuron tactile receptive field, the response appeared. In other words, other’s body space was matched to the monkey’s one. This result indicates a possible way for encoding others’ peripersonal space, and might extend the role of the mirror mechanism in action understanding to others individuals’ actions aimed at themselves. However, in this study the motor responses of the neurons have not been recorded, and it is impossible to tell whether these neurons actually were mirror neurons, nonetheless, it is known that area VIP is strictly connected with premotor area F4 (Matelli et al., 1986; Barbas and Pandya, 1987; Cavada and Goldman-Rakic, 1989; Andersen et al., 1990; Lewis and Van Essen, 2000), where peripersonal space is encoded in terms of reaching movements (Gentilucci et al., 1988; Fogassi et al., 1996; see Graziano, 2006). It is plausible, therefore, that the visual responses actually represent potential motor acts directed toward specific body parts (Gentilucci et al., 1988; Fogassi et al., 1996).

A subsequent study investigated a further important aspect of the observed actions, that is the view-dependence of the visual responses of mirror neurons (Caggiano et al., 2011). To this purpose the monkey was required to observe movies showing the same grasping motor act from three different points of view: in the subjective perspective (0°), and in two types of third-person views, a lateral (90°) and a frontal (180°) one. Among the tested mirror neurons, about three-fourth showed a preference for one of the vantage points, encoding in equal percentage the three different perspectives employed. On the base of these results it has been proposed that *view-independent* mirror neurons encode the goal of the observed motor act irrespective of the visual details of the scene, while *view-dependent* mirror neurons provide a link between the goal of the motor act and its pictorial aspects. Similarly to the mirror neurons modulated by peripersonal or extra-personal space, *view-dependent* mirror neurons could be important for preparing an adequate response to the observed action. These neurons could be part of a neural circuit of the “social brain” coding the spatial relations at the roots of basic social interactions.

In all the reported studies on mirror neurons, actions were unidirectional and non-interactive, while in nature, most often, monkeys interact within complex social environments in which different individuals share the same social space. Here we refer to social interactions as the acts of two or more individual taking into account of other’s actual or potential actions or intentions. By combining a motion capture system with chronic multielectrode recording from different cortical areas (multi-dimensional recording), Fujii et al. (2007, 2008) were able to study the neural activity from monkeys’ parietal and premotor cortex in a social context. When two monkeys were sitting one close to the other, and could reach for and grasp food without interacting, parietal activity resulted to be strongly tuned to the use of the arm contralateral to the recorded hemisphere. However, when the food was put in a shared space and a social conflict emerged between the monkeys, the neurons developed different combinations of preferences to self and other motion (Fujii et al., 2007). This evidence indicates that parietal neurons can recognize social cues and provide other areas with a neural code modulated by social information. The same authors also described the responses of premotor and parietal neurons during the observation of action in a task in which two monkeys were present, but could not interact (Fujii et al., 2008). During action execution both premotor and parietal neurons showed a strong preference for actions performed with the arm contralateral to the recording hemisphere (right arm). During the observation of the other monkey action, the premotor neurons preferred the other monkey right arm movements, while the parietal neurons typically lost this laterality preference, showing a wider spectrum of combinatorial responses to own/other right/left responses. Indeed, the arm used (right or left) in a specific context (position of the food on the right or left side) is very relevant to understand the intention of an action. Accordingly, the authors propose that the premotor neurons code information on action’s agent and effector as primitives of action recognition within the mirror network, while parietal neurons represent the social space and participate in recognizing others’ actions with respect to one’s own actions (Fujii et al., 2008).

The mirror mechanism can enable to understand others’ action, but in this process, the sense of agency appears to be revoked: the neurons are active both when I act and when I see someone else acting, without moving. The lack of synchronicity between the vision of an action and the somato-motor signals related to action execution probably represents the crucial information for attributing the action to self or others (see Wolpert and Ghahramani, 2000; Gazzola and Keysers, 2009; Pitti et al., 2009). Interestingly, different studies described neurons activated by the observation of actions but not discharging during action execution, in the premotor, parietal and temporal cortex (Perrett et al., 1989; Gallese et al., 1996; Fujii et al., 2008). It was proposed that these neurons, by separating visual and somato-motor information, could play a role in the attribution of agency to others (Fujii et al., 2008). The same authors also propose that this function does not rely on single cortical areas, but on a larger cortical network capable of integrating visual and somato-motor informations. Possible networks involved in action observation and participating to this function are described in the following section.

### Anatomo-Functional Mirror Pathways

Among the cortical areas involved in the processing of visual information, those located in the superior temporal sulcus (STS) are generally considered as the fundamental node coding biological information. In fact, STS contains neurons coding visual information about eye/gaze direction, body/limbs orientation and movement, facial expressions, and biological motion (Bruce et al., 1981; Perrett et al., 1989; Puce et al., 1998; Pelphrey et al., 2003; Tsao and Livingstone, 2008). These features are among the most relevant aspects needed by an individual to interpret others’ behavior, and, for this reason, STS is generally considered as the initial stage in processing social cues. To date, very few studies have been done to elucidate how the mirror system interplays with STS or with other parts the “social brain.” Notwithstanding the paucity of available data on this topic, a few studies are unveiling possible pathways by which social information can reach mirror neurons’ computation.

A recent study employed fMRI technique in the monkey to identify the frontal areas active during the observation of motor acts (Nelissen et al., 2005). By using anatomically defined regions of interest, the authors found that viewing videos showing a hand grasping an object activates F5a, F5p, and the prefrontal areas 45A, 45B, and 46. When the video showed the whole individual grasping an object, and not only a hand, the activation also involved F5c. This indicates that there are multiple representations of others’ actions in the monkey frontal cortex and that they can be sensitive to different features. F5a, F5p and the prefrontal areas appear to encode the action as such, while F5c action representation is more centered on the agent doing the action. This result has two important implications: first, a context-dependent processing of the act of grasping is taking place in F5c; second, an input coming from areas processing the visual features of the scene (like STS), makes its way to the premotor cortex.

A subsequent anatomo-functional study made by Nelissen et al. (2011) investigated how visual information about action can reach the frontal areas, and concentrated on STS and posterior parietal cortex fMRI activation in the monkeys during the observation of grasping acts. They also correlated functional with connectional data obtained by means of neural tracers injections. The employed videos activated areas in the lower and upper banks of STS, and in the IPL. An analysis based on regions of interest showed that grasping observation activates stronger than control conditions three IPL areas (PFG, on the cortical convexity, AIP and LIP in the lower bank of intraparietal sulcus) and five STS regions (MT/V5, LST, and LB2 in the lower bank, FST in the fundus and STPm in the upper bank). Note that a recent electrophysiological study directly demonstrated the presence of mirror neurons in the AIP area (Pani et al., 2014).

In order to assess which of the STS areas active during action observation are actually connected with the mirror areas, retrograde tracers were injected in the parietal nodes of the mirror system (AIP and PFG). After AIP injections, a widespread STS labeling were found, but the most consistent labeling in all cases was in the lower bank sector LB2 and in the inferotemporal cortex near the lip of STS. Injections in PFG resulted in consistent labeling in STS upper bank sectors MSTd, STPm, and UB1. Note that, of them, only STPm was found to be specifically active during action observation.

**FIGURE 5:**
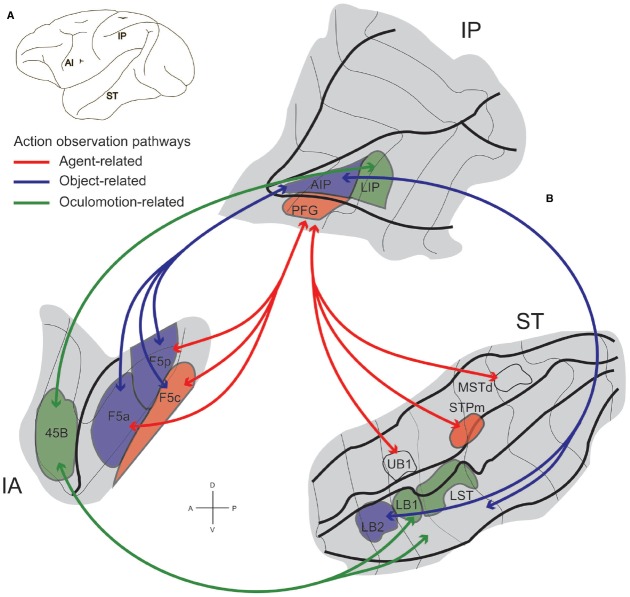
**STS–IPL-F5 grasping observation networks in the monkey. (A)** Lateral view of a macaque brain showing locations of three regions involved in action observation: inferior arcuate sulcus (IA), Intraparietal sulcus and inferior parietal lobule (IP), and superior temporal sulcus (ST). **(B)** Flattened representation of inferior arcuate, intraparietal and superior temporal sulci. Visual information on observed actions can be sent from STS through parietal cortex to premotor area F5 along two functional routes: a STPm–PFG–F5c, agent-related action observation pathway (red lines) and a LB2–AIP–F5a/p object-related action observation pathway (blue lines). Visual information from STS can also reach parietal and prefrontal areas involved in oculomotion, through the LB1/LST-LIP-45B oculomotion-related action observation pathway (green lines). The arrows specify the functional routes. For abbreviations, see text. Modified from Nelissen et al. (2011).

This integrated anatomo-functional approach led to the identification of two functional pathways involved in action observation linking STS, IPL, and PMv (Figure 5, red and blue). One links STS sector STPm with parietal area PFG that, in turn, is connected with premotor area F5c. The other pathway connects LB2 with AIP that, in turn, is connected with F5a and F5p. Both routes process information necessary for understanding the observed motor act, but each provides a different type of information, possibly playing a specific role in understanding the intention underlying it. In particular, the STPm-PFG-F5c pathway is more concerned with the *agent doing the action*, while the LB2-AIP-F5a/p one with the *details of grip* and *object identity* (Nelissen et al., 2011).

These pathways show that the parietal regions containing mirror neurons have a direct access to STS information about biological motion, crucial for coding the observed agent’s actions and intentions. This direct access implies that PFG/AIP would be a first node where the neural codes for grasping and for social information—like gaze, head, body, or limb orientation or direction—are integrated in a common motor representation that becomes available for mirroring others’ actions. This integrated code in which grasping is linked with social information would then be sent to F5 in the premotor cortex.

A further pathway links area 45B in the prearcuate cortex with LST and LB1 in the lower bank of STS, and LIPa in the lower bank of intraparietal sulcus (Figure 5, green). Note that monkey area 45B is known to be part of the oculomotor system, probably representing the gateway of highly integrated prefrontal and orbitofrontal information to this system (Moschovakis, 2004; Gerbella et al., 2010). It was proposed that the LST/LB1-LIP-45B pathway could play a role in oculomotor control during action observation (Gerbella et al., 2010; Nelissen et al., 2011). Indeed, gaze behavior mirroring has been found in LIP of macaque monkeys where a sub-population of neurons discharge both when monkeys direct their gaze in a given direction and when they look at a static image of another monkey having the gaze oriented in that same direction (Shepherd et al., 2009). The areas of the LST/LB1-LIP-45B pathway are activated by action observation (Nelissen et al., 2011), but there is no evidence of the presence of grasping mirror neurons in any of them. So the question remains: where does the integration of information related to grasping and gaze direction occurs? This question is even more relevant considering the importance of parsing others’ gaze direction for deciphering their intention (see Klein et al., 2009). The existence of this pathway raise the possibility that STS information about biologically or socially relevant gaze targets reaches oculomotor areas LIP and 45B. However, “oculomotor mirroring,” confirmed in LIP, remains untested in prefrontal cortex. One could reasonably expect to find a population of neurons mirroring gaze behavior in area 45B. Note that in this pathway, gaze information would still be segregated from the one coding grasping. The anatomical pathways through which gaze mirroring would reach the parietal and premotor areas of grasping mirroring still remain to be described, but probably include the prefrontal cortex (see below).

Summing up, the STS information about social cues deriving from biological motion analysis could reach the mirror system directly (STPm-PFG-F5c and LB2-AIP-F5a/p pathways) or indirectly through an “oculomotor” mirroring system (LST/LB1-LIP-45B pathway). These hypotheses are not mutually exclusive, and thus far, there is no data directly confirming any of them. However, there are indirect behavioral and electrophysiological evidence suggesting that information about gaze direction and action observation converge and probably become integrated in the same neural code. The behavioral data comes, from a well-known human study showing that subjects display the same gaze pattern when performing a grasping action and when observing another individual performing the same action (Flanagan and Johansson, 2003). The electrophysiological evidence comes from preliminary data showing that in monkeys area F5, the activity of some mirror neurons is modulated by the gaze direction of the observed agent (Coudé et al., 2013).

### Prefrontal Cortex and Mirror Network

Functional MRI studies demonstrated that prefrontal area 46 is involved in action observation (Nelissen et al., 2005) and motor-related activity in the ventral prefrontal cortex has been described (Tanila et al., 1992; Hoshi et al., 1998; Rozzi et al., 2011). More recently, connectional studies on the ventral prefrontal cortex indicated that a specific sector of ventral area 46 (rostral part of 46VC) and area 12 (intermediate 12r) is connected with different nodes of the mirror pathways (Borra et al., 2011; Gerbella et al., 2013). These nodes include rostral premotor area F5a, IPL areas PFG and AIP and a sector of the ventral bank of rostral STS sector, which overlaps with the fMRI sites activated by action observation. Altogether, these evidence indicate that certain parts of the prefrontal cortex might be considered as actual components of the mirror system, but electrophysiological confirmation of this hypothesis is still lacking. A possible role of the prefrontal cortex within the mirror system, could be to provide the motor representations of the parietal and motor areas with mnemonic and contextual information. The mirror system access to this kind of information could, for instance, allow action understanding when the target object is not actually visible during the unfolding of a grasping action (Umiltá et al., 2001). It could also enable intention understanding by retrieving the meaning of contextual cues previously associated to specific actions (Fogassi et al., 2005). In addition, the ventral prefrontal cortex could provide the parietal and premotor cortex with social contextual cues. Such cues would consist in information about gaze direction or body part orientation, as elaborated in STS. Interestingly another sector of ventral prefrontal cortex (caudal part of 46VC) is strongly connected with frontal and parietal oculomotor areas, as well as with the STS and the other sectors of area 46. This pattern of connections could represent a pathway, though indirect, linking the oculomotor system with the mirror system.

Note that the connections between prefrontal areas and mirror areas are bidirectional. This implies that, from the one side, the VLPF can modulate mirror neuron activity by sending mnemonic and contextual information, from the other, the parieto-premotor areas could provide the prefrontal cortex with motor representations of action goals. Thus, a further role of the prefrontal cortex could consist in recombining the observed motor acts, captured by the parietal and premotor nodes of the mirror system, to produce an action fitting the observed model, allowing imitative learning, as suggested by studies on humans (Buccino et al., 2004, see below). Further studies will have to verify these hypotheses and assess the specific contribution of the prefrontal areas, classically considered to exert a top-down control on sensory and motor areas, to the mirror system.

## The Mirror System in Humans, An Anatomo-Functional Perspective

The mirror system is thought to constitute a fundamental part of the vertebrate motor system and has presumably been conserved and adapted through different species, including humans. The previous sections outlined its circuitry and functions in the monkey. Technical and ethical limitations precludes to reach a similar level of details in the description of the human mirror system. However, studies using non-invasive techniques like brain imaging, TMS and EEG/MEG have yield evidence that a mirror system exists in humans (Fadiga et al., 1995; Grafton et al., 1996; Rizzolatti et al., 1996b; Pfurtscheller and Neuper, 1997; Hari et al., 1998; Cochin et al., 1999; Grèzes et al., 1999; Nishitani and Hari, 2000, 2002; Buccino et al., 2001; Gangitano et al., 2001; Perani et al., 2001; Maeda et al., 2002; Muthukumaraswamy and Johnson, 2004; Iacoboni et al., 2005; Oberman et al., 2005; see Pineda, 2005; Rizzolatti et al., 2014).

In EEG studies, Mu waves are detected in the 8–13 Hz frequency range and are thought to be the result of synchronous discharges by resting neurons in the sensorimotor region of the brain (see Kuhlman, 1978; Anderson and Ding, 2011). Mu rhythm suppression occurs during motor preparation, action execution (Neuper et al., 2006), but also during mental imagery and action observation (Cochin et al., 1999; see Pineda, 2005). Brain imaging studies demonstrated a consistent pattern of cortical activity during action observation, involving a network of several brain regions (see Caspers et al., 2010). This action observation network includes Brodmann’s areas 44/45, lateral dorsal premotor cortex, supplementary motor area, primary somatosensory cortex, superior parietal lobule, intraparietal cortex, rostral inferior parietal lobule, posterior middle temporal gyrus at the transition to visual area V5, and fusiform face area/fusiform body area.

Interestingly, this human mirror network largely overlaps with the monkey one (IPL, PMv, and caudal part of inferior frontal gyrus). However, various other areas are active in humans during action observation. The only one description of a single neuron mirroring mechanism was provided by Mukamel et al. (2010), recording from areas not belonging to the classical mirror system. The larger number of areas involved in action observation in humans could depend on several factors. First, most of monkey studies have been carried out by means of single neuron recording. This technique is the only one capable of demonstrating the presence of mirror neurons, but lacks the possibility to explore large brain regions at the same time. Thus, it is likely that the monkey mirror system has not yet been fully mapped. This hypothesis is supported by ^14^C-deoxyglucose autoradiography experiments in monkeys showing that further regions beyond the classical mirror areas—including superior parietal, somatosensory and primary motor areas—are activated by action observation (Evangeliou et al., 2009; Raos et al., 2014), although the actual presence of mirror neurons in these areas is still to be confirmed. A second hypothesis is that the mirror system in humans have expanded to additional cortical areas, probably acquiring new functions. A third possibility is that the brain activation evidenced by brain imaging studies during action observation could be related to different aspects of visual processing or to motor preparation, and be independent on the actual presence of mirror neurons. To our knowledge, none of these hypotheses has been ultimately demonstrated. An interesting attempt has been done by Gazzola and Keysers (2009) in a recent fMRI study aimed at identifying the brain regions activated by both action observation and action execution, and thus, likely containing mirror neurons. The single-subject analysis of unsmoothed fMRI data allowed the authors to identify the voxels shared between action observation and action execution in the classical IPL-PMv circuit, but also in the middle cingulate, dorsal premotor, somatosensory, superior parietal, and middle temporal cortex. The activation of areas not belonging to the classically described parieto-premotor mirror circuit could reflect sensory predictions from internal models (Wolpert and Ghahramani, 2000; Gazzola and Keysers, 2009). This process would complete and enrich the information about others’ actions encoded by the classical mirror system. Further studies on action observation and execution conducted in human and monkey by means of brain imaging and electrophysiological techniques will be important to demonstrate the presence of mirror neurons in the human cortex and to test and disentangle between the different hypotheses proposed above.

The mirror mechanism, besides being involved in action understanding, could also play a role in learning by imitation. Buccino et al. (2004) specifically investigated this issue by means of fMRI. In this study, naive participants were required to observe images depicting the hand of an expert guitarist playing chords and to imitate them after a delay. Action observation, as expected, activated IPL, PMv and the pars opercularis of the inferior frontal gyrus. Noteworthy, these areas together with the prefrontal cortex (area 46 in the middle frontal gyrus) and the anterior mesial cortex were active during the delay phase preceding movement execution. The authors proposed that area 46 could recombine the observed motor acts, captured by the parieto-premotor mirror system in order to produce an act fitting the observed model.

### Others’ Actions in Their Social Context

As mentioned above, the mirror mechanism in monkeys is considered to be involved in coding others’ actions in their social context (Fujii et al., 2007, 2008; Ishida et al., 2010; Visco-Comandini et al., 2015). The term “social context” encompasses a wide spectrum of settings and can refer to complex interactions, especially in human societies. Whereas some forms of human social interactions appear to be unique in their complexity, other forms are more basic and are probably shared with other primates (see Tomasello and Call, 1997). It is thus likely that the same mirror mechanism is involved in the most basic social interactions in different primate species. Human brain imaging and TMS data seems to support this idea. Indeed, it has been showed that areas pertaining to the mirror system are more strongly activated when subjects performed complementary actions rather than when they performed the same action as the one observed (Newman-Norlund et al., 2007). TMS data by Sartori et al. (2011) also point in the same direction and demonstrate that depending on the context, motor-evoked potentials can reflect the observed movement or an appropriate complementary movement. In this experiment, when an object was present and the observer was implicitly required to act upon the object in response to the observed action, a shift from symmetrical motor resonance to complementary activations of hand muscles was observed. Thus, action observation does not inevitably lead to symmetrical motor facilitation, that could be useful for imitation, but could also play a role in successfully performing attuned joint actions.

Human data also showed that intentions and social contexts affect kinematics, and conversely kinematics and contexts affect intention understanding. The kinematics of a grasping act differ depending on the final goal of the action (e.g., grasping to move, to throw or to pass, see Becchio et al., 2012). On the other hand, the context provides clues for understanding the intention underlying the observed motor act, and is known to modulate the activity of the caudal sector of IFG, during action observation (Iacoboni et al., 2005). This mean that, also in humans the mirror system is involved in intention coding. In addition, it has been showed that reaching toward an object and grasping it either to move it from one spatial location to another or to place it into the hand of a partner yield different kinematics (Becchio et al., 2010; for similar results see also Mason and Mackenzie, 2005; Meulenbroek et al., 2007). Interestingly, the observation of social movements evokes an activation stronger than non-social ones within mirror areas, including the IFG and the IPL (Becchio et al., 2012). These finding demonstrates that areas within the mirror system are sensitive to very subtle differences in the observed action’s kinematics. Most importantly, it suggests that mirror areas in humans are more responsive to social than non-social movements. Similarly, Mu rhythm suppression has been shown to be greater for social interactive actions than for non-social actions (Oberman et al., 2007).

Altogether, these data suggest that during social interaction, human agents decipher the goal of others’ ongoing action and integrate it into their own action planning, eliciting different potential complementary responses. Thus, the mirror mechanism, being tuned to social actions, besides its known role in motor cognition, is likely involved in social cognition.

## Conclusion

This review was an attempt to outline an updated view of the organization of the neural bases of grasping. Our knowledge of the motor system hinges on a multidisciplinary approach applied to the macaque monkey model. Obvious technical and ethical limitations preclude the application of such method to humans. However, clear homologies have been established between the motor systems of the two species. The basic mechanisms underpinning grasping actions are very likely shared among primates and humans. Among these mechanisms is the neuronal coding of movements in terms of motor goals and the mirror mechanism, allowing to retrieve these goals during action observation. The latter is an in-built motor resonance mechanisms, deemed to be at the core of action understanding. We believe that such neural coding, pertaining to the motor system and originally evolved for guiding behavior, has later become a fundamental component on which social cognition was constructed. However, the possible role of other processes, for instance involving mentalizing, should not be downplayed and could work in parallel with the mirror system.

The mirror system is not only reflecting what another individual is doing, but also integrates contextual aspects like spatial cue, gaze direction or kinematic parameters. We discuss how this process of internal simulation is at the bases of action and intention understanding in monkeys and humans. Human data also yield a further fundamental function of the mirror mechanism: allowing the preparation of appropriate complementary responses to the observed actions. This latter process could explain how two individuals become attuned to cooperate in a joint action. It also underlines the flexibility of the mirror system.

Complex functions cannot depend on a single brain region but are rather the results of several areas linked together by cortical connections, and forming functionally specialized networks. The grasping execution/observation system is no exception. Clearly, specific sets of temporal, parietal and motor areas contribute to different aspects of the mirror system functions. This suggests that the mirror neuron network extends probably beyond the motor system to include other cortical sectors. A deeper investigation of the role of these putative nodes of the mirror system, and especially of those located in the prefrontal cortex, will be crucial for defining the relationships between the classical mirror circuit and other centers possibly exerting a top-down control on them. This, in turn, will prompt a better understanding of how information about the social context can influence our comprehension of actions and intentions, and shape our own motor programs.

### Conflict of Interest Statement

The authors declare that the research was conducted in the absence of any commercial or financial relationships that could be construed as a potential conflict of interest.
